# Superior Mesenteric Vein and Portal Vein Thrombosis in an Immunocompetent Patient With Acute Cytomegalovirus Infection

**DOI:** 10.7759/cureus.107837

**Published:** 2026-04-27

**Authors:** Amro Abdellatief, Pinky Bai, Hetu Patel, Sameer Krishna Prasad Garlapati, Angesom Kibreab

**Affiliations:** 1 Gastroenterology and Hepatology, Howard University Hospital, Washington, DC, USA; 2 Internal Medicine, Howard University Hospital, Washington, DC, USA; 3 Medicine, Howard University Hospital, Washington, DC, USA; 4 Internal Medicine, Andhra Medical College, Visakhapatnam, IND; 5 Gastroenterology and Hepatology, Howard University College of Medicine, Washington, DC, USA

**Keywords:** acute cmv infection, infection, portal vein thrombosis (pvt), smv thrombosis, splenic infarct

## Abstract

Cytomegalovirus (CMV) infections are common worldwide, with clinical manifestations varying from asymptomatic infections in healthy people to life-threatening end-organ dysfunction in immunocompromised patients. Acute CMV infection has been associated with venous thrombosis in immunocompetent patients. We present a rare case of acute superior mesenteric vein (SMV) and portal vein thrombosis in an immunocompetent patient with acute CMV infection. A 28-year-old patient with a past medical history of obesity presented with two days of sharp, 9/10 left upper quadrant (LUQ) pain that radiated to the back, associated with subjective fevers, nausea, and loose stools. The patient was tachycardic, febrile, and tachypneic. Physical examination revealed LUQ tenderness to superficial and deep palpation. Laboratory findings showed leukocytosis with lymphocytic predominance as well as transaminemia. CT of the abdomen/pelvis revealed SMV and portal vein thrombosis, hepatomegaly, and splenomegaly with multiple splenic infarcts. The thrombophilia workup was negative. CMV testing showed positive IgM. CMV polymerase chain reaction was elevated at 1,222 log IU/mL. He was started on ganciclovir and improved clinically. He was discharged on oral ganciclovir and warfarin. This case report emphasizes that, in an immunocompetent patient presenting with acute deep vein thrombosis at unusual sites, a negative thrombophilia workup, and after excluding other risk factors, CMV infection as a triggering etiological factor should be considered.

## Introduction

Cytomegalovirus (CMV) infections are common, with over 59% of the population older than six years of age having been exposed to it [1]. Clinical manifestations vary from asymptomatic infections in healthy people to life-threatening end-organ dysfunction in immunocompromised patients [1]. Studies have shown that acute CMV infection has been associated with venous thrombosis in immunocompetent patients [2]. We present a rare case of acute superior mesenteric vein (SMV) and portal vein thrombosis in an immunocompetent patient with acute CMV infection.

This article was previously presented as a meeting abstract at the 2025 ACG conference in Phoenix, AZ, Annual Scientific Meeting on October 27, 2025.

## Case presentation

A 28-year-old patient with a past medical history of obesity presented with two days of sharp, 9/10 left upper quadrant (LUQ) pain that radiated to the back, associated with subjective fevers, nausea, and loose stools. The patient denied any recent travel, sick contacts, or any other known medical conditions. He worked in construction and denied consumption of alcohol, tobacco, or illicit drugs. He took no medications or over-the-counter herbal supplements.

On arrival, vitals showed tachycardia to 140s beats per minute, febrile to 102.5°F, tachypneic to the 20s with normal oxygen saturation on room air. Physical examination revealed he was in no acute distress but had LUQ tenderness to superficial and deep palpation. Laboratory investigations (Table 1) showed leukocytosis with white blood cell count of 18,400 cells/mm³ (normal: 4,000-11,000 cells/mm³) with predominant lymphocytes of 10,650 cells/mm³ (normal: 1,000-4,800 cells/mm³), elevated creatinine of 1.26 mg/dl (normal: <1.2 mg/dL), elevated transaminases with alanine aminotransferase of 95 IU/L (normal: <40 IU/L), aspartate aminotransferase of 61 IU/L (normal: <40 IU/L), and alkaline phosphatase of 146 IU/L (normal: 30-120 IU/L) with normal total bilirubin. HIV, T-spot, and acute hepatitis workup were negative. Blood cultures, stool cultures, Clostridioides difficile, and stool ova and parasites were negative. However, fecal leucocytes returned positive. Flu, respiratory syncytial virus, and COVID-19 tests were negative. Urinalysis showed no evidence of a urinary tract infection.

**Table 1:** Laboratory values with normal ranges.

Laboratory marker	Patient’s value	Normal range
White blood cell count	18,400 cells/mm³	4,000–11,000 cells/mm³
Lymphocyte count	10,650 cells/mm³	1,000–4,800 cells/mm³
Creatinine	1.26 mg/dL	<1.2 mg/dL
Alanine aminotransferase	95 IU/L	<40 IU/L
Aspartate aminotransferase	61 IU/L	<40 IU/L
Alkaline phosphatase	146 IU/L	30–120 IU/L

**Figure 1:**
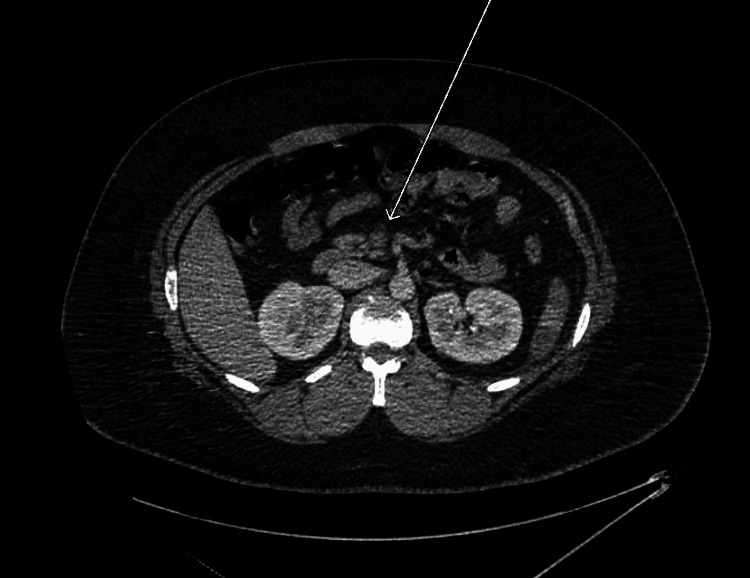
Axial CT image reveals a filling defect within the splenic vein (arrow), consistent with mesenteric vein thrombosis.

**Figure 2:**
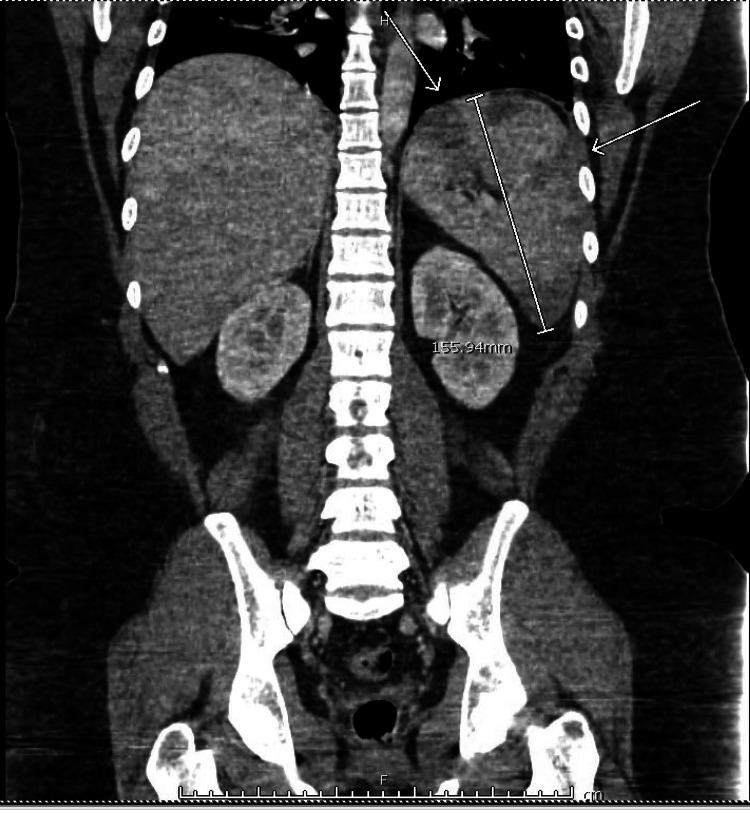
Coronal CT image shows a significantly enlarged spleen (white arrow) with multiple splenic infarcts.

**Figure 3:**
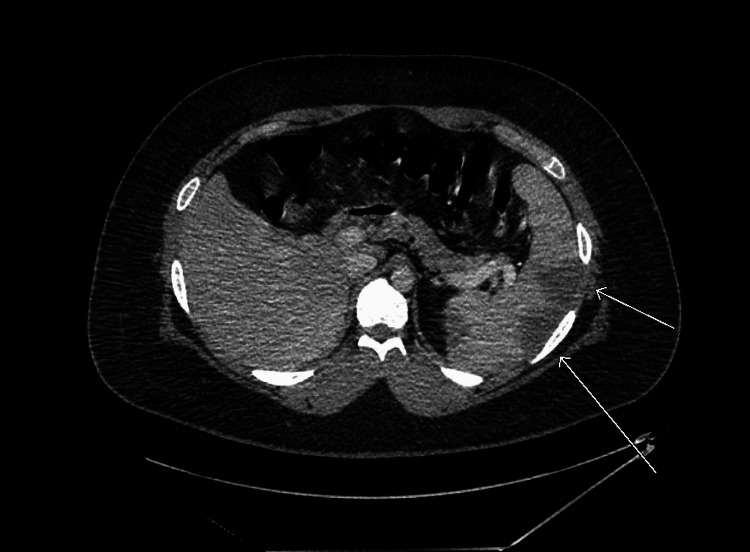
Axial CT image demonstrating multiple splenic infarcts (white arrows).

Duplex ultrasound of his lower extremities was negative for deep vein thrombosis, while CT angiography of the chest was negative for pulmonary embolus. CT of the abdomen/pelvis revealed SMV thrombosis (Figure 1), portal vein thrombosis (Figure 2), as well as splenomegaly (Figure 1) with multiple splenic infarcts (Figures 1, 3). He was promptly initiated on heparin infusion.

Gastroenterology, hem-oncology, cardiology, and infectious disease (ID) teams were consulted. Thrombophilia workup, including factor V Leiden, Protein C and S, *JAK2* mutation, paroxysmal nocturnal hemoglobinuria flow cytometry, antiphospholipid antibodies, prothrombin gene analysis, double-stranded DNA antibody, beta-glycoprotein antibody, antinuclear antibody, anticardiolipin antibody, and lupus anticoagulant, was all negative or within the normal range. He underwent diagnostic esophagogastroduodenoscopy for abdominal pain and to rule out esophageal varices, which showed a prominent ampulla of Vater and superficial punctate lesions in the duodenal bulb. Gastric biopsies were negative for *Helicobacter pylori*, and biopsies of the duodenal lesions showed no histopathological abnormality. He was also scheduled at a later date for a diagnostic colonoscopy for suspicion of infectious causes of his persistent loose stools. ID recommended testing for syphilis, which returned negative. CMV testing showed positive IgM. CMV polymerase chain reaction was elevated at 1,222 log IU/mL. He was started on ganciclovir and improved clinically. He was discharged on oral ganciclovir and warfarin with instructions for outpatient follow-up.

## Discussion

A meta-analysis has shown that there have been nearly 100 cases of CMV-associated thrombosis in the medical literature [3]. In the study, the incidence of thrombosis among hospitalized patients with acute CMV infection was 6.4%, and the incidence of acute CMV infection among hospitalized thrombosis patients was 1.9-9.1%. Most (n = 64; 65.9%) reported patients were immunocompetent [3]. This was similar to our case, as the patient was an otherwise healthy, immunocompetent young male patient who developed acute portal vein thrombosis and mesenteric vein thrombosis in the setting of acute CMV infection. A prospective study concluded that acute CMV infection might be associated with an increased short-term venous thromboembolism (VTE) risk [4]. This study concluded that CMV IgM seropositivity was independently associated with VTE appearance [4]. This finding was similar to that of our patient, who developed acute SMV and portal vein thrombosis while in the acute phase of CMV infection.

A systematic review showed that in immunocompetent patients, splanchnic vein thrombosis (SVT) and splenic infarction have been suggested to represent approximately half of the published cases of CMV-associated VTE [5], which was similar to our patient. Another study from the Netherlands demonstrated two cases of acute CMV infection in immunocompetent patients: one developed acute portal vein thrombosis and the other developed acute pulmonary embolism [6]. Another case report described a 25-year-old, immunocompetent female patient with acute CMV infection who developed acute cerebral venous sinus thrombosis. She responded well to anticoagulation with warfarin [7]. This was similar to our patient who recovered from the CMV infection with ganciclovir and warfarin for thromboses. Another study demonstrated a 30-year-old male patient who, in the setting of acute CMV infection, developed thrombosis in the right branch of the portal vein [8]. Another case of an immunocompetent 24-year-old lady who developed symptomatic portal vein thrombosis secondary to acute CMV infection highlights the similarities with our case [9].

A retrospective study demonstrated that within one year of CMV infection, 38 of 379 (10.0%) patients developed VTE in the study group compared to 41 of 1,334 (3.1%) patients in the seropositive control and 37 of 1,249 (3.0%) in the seronegative control [10]. These studies indicate that acute CMV infection in young, relatively healthy, and immunocompetent patients is a risk factor for developing acute venous thrombosis in unusual sites. This case report should prompt further studies into the relationship between acute CMV infection and venous thrombosis. It should also prompt further studies into the consideration of initiating a screening protocol for VTE in hospitalized patients with acute CMV infection.

## Conclusions

This case highlights acute CMV infection as an important diagnostic consideration in young, otherwise healthy patients presenting with venous thrombosis at unusual sites, particularly when accompanied by fever, lymphocytosis, splenomegaly, and mild hepatitis. Although causality cannot be definitively established from a single case, this report adds to the growing literature suggesting an association between acute CMV infection and SVT in immunocompetent adults.

## References

[1] Gupta M, Shorman M (2024). Cytomegalovirus. https://www.ncbi.nlm.nih.gov/books/NBK459185/.

[2] Yildiz H, Zech F, Hainaut P (2016). Venous thromboembolism associated with acute cytomegalovirus infection: epidemiology and predisposing conditions. Acta Clin Belg.

[3] Justo D, Finn T, Atzmony L, Guy N, Steinvil A (2011). Thrombosis associated with acute cytomegalovirus infection: a meta-analysis. Eur J Intern Med.

[4] Paran Y, Shalev V, Steinvil A (2013). Thrombosis following acute cytomegalovirus infection: a community prospective study. Ann Hematol.

[5] Bertoni M, Squizzato A, Foretic M, Zanieri S, Di Natale ME (2018). Cytomegalovirus-associated splanchnic vein thrombosis in immunocompetent patients: a systematic review. Thromb Res.

[6] Neppelenbroek SI, Rootjes PA, Boxhoorn L, Wagenaar JF, Simsek S, Stam F (2018). Cytomegalovirus-associated thrombosis. Neth J Med.

[7] Martin AJ (2023). Cerebral venous sinus thrombosis secondary to acute cytomegalovirus infection. BMJ Neurol Open.

[8] Puccia F, Lombardo V, Giannitrapani L, Licata A, Mazzola G, Soresi M (2017). Case report: acute portal vein thrombosis associated with acute cytomegalovirus infection in an immunocompetent adult. J Ultrasound.

[9] Galloula A, Rossi A, Gautier V, Minozzi C, Messas E, Mirault T (2014). [Portal vein thrombosis associated with an acute cytomegalovirus infection]. J Mal Vasc.

[10] Kelkar AH, Loc BL, Tarantino MD, Rajasekhar A, Wang H, Kelkar M, Farrell J (2020). Cytomegalovirus-associated venous and arterial thrombotic disease. Cureus.

